# Environmental hazard assessment for polymeric and inorganic nanobiomaterials used in drug delivery

**DOI:** 10.1186/s12951-019-0489-8

**Published:** 2019-04-16

**Authors:** Marina Hauser, Guangyu Li, Bernd Nowack

**Affiliations:** Empa, Swiss Federal Laboratories for Materials Science and Technology, Lerchenfeldstrasse 5, 9014 St. Gallen, Switzerland

## Abstract

**Background:**

The increasing development and use of nanobiomaterials raises questions about their potential adverse effects on the environment after excretion and release. Published ecotoxicological data was searched for five polymeric nanobiomaterials [chitosan, polylactic acid (PLA), polyacrylonitrile (PAN), polyhydroxyalkanoates (PHA), and poly(lactic–glycolic acid) (PLGA)] and one inorganic nanobiomaterial [hydroxyapatite (HAP)] to evaluate the environmental hazards for freshwater and soil using a meta-analysis. If enough data was available, a probabilistic species sensitivity distribution (pSSD) and from this a predicted no effect concentration (PNEC) was calculated. If only one data point was available, a PNEC was calculated based on the most sensitive endpoint. Each material was classified either as “nano” or “non-nano”, depending on the categorization in the original articles. When the original article specified that the material consisted of nanoparticles, the material was classified as nano; when nothing was mentioned, the material was classified as “non-nano”.

**Results:**

For PLA, PHA and PLGA, no published data on ecotoxicity was found and therefore no hazard assessment could be conducted. In soils, HAP was found to have the lowest PNEC with 0.3 mg/kg, followed by PAN and chitosan. In freshwater, chitosan was found to have the lowest PNEC with 5 µg/l, followed by nano-chitosan, HAP and PAN.

**Conclusion:**

Compared with other common pollutants, even the most sensitive of the selected nanobiomaterials, chitosan, is less toxic than engineered nanomaterials such as nano-ZnO and nano-Ag, some common antibiotics, heavy metals or organic pollutants such as triclosan. Given the current knowledge, the nanobiomaterials covered in this work therefore pose only little or no environmental hazard.

**Electronic supplementary material:**

The online version of this article (10.1186/s12951-019-0489-8) contains supplementary material, which is available to authorized users.

## Background

During the past decade, nanobiomaterials (NBMs) have become increasingly important for the use in biomedical engineering and pharmaceutics [1]. At present, one of the hot topics in nanomedicine is the use of NBMs for drug delivery. A biomaterial is any material designed to interact with biological systems for a medical purpose. The International Standards Organization (ISO) has defined a nanomaterial as a material with one, two or three external dimension in the nanoscale (1–100 nm), whereas a nanoparticle on the other hand is defined as a particle with all three external dimensions smaller than 100 nm [2, 3].

Inorganic nanomaterials have commonly been used in nanomedicine. Their relatively simple generation and surface modification as well as biocompatibility make gold (Au) nanoparticles attractive for utilization in medical imaging or cancer detection and treatment [4–6]. Silver (Ag) nanoparticles are applied as coatings for indwelling catheters, antibacterial agents, wound dressing, orthopedic implants, and tissue-engineered scaffolds. Iron oxide (Fe_3_O_4_) nanoparticles are used for bioimaging, photothermal therapy, and biosensing [7]. Hydroxyapatite (HAP) belongs to the group of calcium phosphates and is a naturally occurring mineral of biological and agricultural importance. For example, human and animal bones are composed of HAP [8, 9]. Various applications of HAP have been explored, including their use in coatings promoting bone regeneration and as drug carriers for antibiotic delivery. Combining HAP with antibiotics is considered very promising and may guarantee bone implant integration without bacterial adhesion [8, 10].

Polymer-based nanomaterials are often used in medical applications due to their high biological safety, good biodegradability, and easy production and modification [11]. Examples of polymeric NBMs are chitosan, polylactic acid (PLA), polyacrylonitrile (PAN), polyhydroxyalkanoates (PHA), and poly(lactic–glycolic acid) (PLGA). Chitosan is a polysaccharide that is found in the exoskeleton of crustaceans and is used for fast wound healing or as a blood clotting agent [12]. PLA has good elastic modulus, thermal formability, and mechanical strength and is therefore used in cartilage regeneration, bone tissue engineering, and cartilage repair. Besides, as PLA helps releasing a drug gradually through slow degradation in vivo, it is used in the formulation of controlled-release drugs [11]. PAN is well known for producing carbon nanofibers with excellent thermal properties, spinability, processability, mechanical stability, and resistance to most chemicals, microorganisms, heat and sunlight. Additionally, PAN fibers have been investigated for their controlled release of various metal nanoparticles like silver or gold nanoparticles together with organic and inorganic additives for antibacterial therapy [13]. PHA is a new class of microbial biopolymers which has attracted great interest from tissue engineers as a potential medical material [14]. PLGA is widely used in nanoparticles, microspheres, pellets, and microcapsules. Drugs encapsulated in PLGA reduce adverse reactions and better accumulate in tumors [11].

Like all other anthropogenic substances, NBMs can find their way into the environment where individuals, populations, communities and ecosystems may be exposed to them in ways that can cause impacts. It is important to distinguish between the presence of a substance with no significant adverse effect and the presence of substances at levels that cause adverse effects. Ecotoxicology is concerned with the relationship between the outputs from human activities and their impacts on ecosystems [15]. Pharmaceuticals and NBMs are expected to behave similarly in the environment. Like pharmaceuticals, NBMs are excreted in urine and feces and so enter the sewage system from where they are eventually discharged into surface waters and distributed throughout the biosphere. A study by the German Federal Environment Agency has reported the detection of a total of 156 pharmaceuticals in different environmental media such as surface water, groundwater and drinking water [16]. Most pharmaceuticals were found in surface waters in a concentration range of 0.1–10.0 μg/l. Worldwide, more than 600 pharmaceutical substances were identified in the environment [17] and 17 pharmaceutical substances were found in each of the United Nations regions [16]. Toxic effects from pharmaceuticals have been shown from a molecular level such as inhibition of cyclooxygenase, up to the population level such as behavioral changes and effects on reproduction [17, 18]. For this reason, the German Umwelt Bundesamt has conducted an environmental risk assessment of 120 human medicinal products with the result that approximately 10% present a notable potential environmental risk [17].

The chemical, physical and biological characteristics of nanomaterials are considerably different from the properties of microscale particles due to their higher surface area over volume ratio [2, 19, 20] and nano-specific properties that arise from quantum or surface effects [21]. Therefore, available experience with existing polymeric or inorganic chemicals regarding human health and environmental safety may not be relevant to NBMs [22]. Besides some nanomaterials may affect the environment less severely than they affect human health, whereas others may be more hazardous for the environment [23]. So even if a NBM is biocompatible and safe for humans, it does not mean the same is true for the environment. These uncertainties regarding the safety of NBMs could hold back their future market growth. Therefore, it is important to evaluate the risk of these materials as they may exhibit adverse effects towards human health or the environment [4, 19].

A first step in an environmental risk assessment is the hazard assessment. The overall hazard to an environmental compartment can be described by the predicted no effect concentration (PNEC), which is the threshold at which no adverse effects are expected on the ecosystem [24]. The PNEC can either be obtained from the lowest observed no-effect concentrations (NOECs) or from statistical extrapolation methods using cumulative species sensitivity distributions (SSDs) [25]. Hazard assessments using SSDs for calculating the PNEC have been published for various engineered nanomaterials (ENMs) [26–29]. Regarding NBMs, only one study evaluating the risk of gold nanomaterials from medical applications has been published so far [30]. No study has been performed yet on the hazard of polymeric or other inorganic nanomaterials used for drug delivery. Therefore, the aim of this work was to conduct a first environmental hazard assessment for the widely-studied polymeric nanobiomaterials chitosan, PLA, PAN, PHA, and PLGA as well as the inorganic NBM HAP based on a meta-analysis of published ecotoxicity studies. If possible, the nanoform of the material was compared to the bulk or dissolved form to identify potential nanospecific toxicity.

## Methods

### Hazard data collection

The environmental hazard literature for chitosan, PLA, PAN, PHA, PLGA and HAP published before October 2017 in journals with an impact factors higher than 2 in 2016 was examined. Additionally, Material Safety Data Sheets from relevant companies were used, although they only contributed to 0.8% (2 data points) of the total database.

Only ecotoxicological effects on survival, growth, reproduction, hatching and changes in significant metabolic processes (such as photosynthesis) were considered [27]. Within one study, only data for one major effect was collected to avoid over-representation a singly study. Minor effects like changes in behavior, coloring, mild biochemical adjustments, or enzyme regulations were excluded. Cytotoxicity studies on in vitro tests with animal cells lines were not included. Additionally, chronic endpoints were preferred over acute endpoints if both were available in the same study. When different particle sources, particle sizes, or culture conditions etc. were tested in the same study, all the different endpoints were considered. Therefore, the data presented later is not restricted to a specific nanomaterial form or particle property (e.g. specific surface coating or surface charge), but rather considers a range of possible nanomaterial characteristics and is thus making the model applicable to a wide range of NBMs. A variety of endpoints were reported and used in the evaluation: MIC (minimum inhibitory concentration), LOEC (lowest observed effect concentration), EC15, EC25, EC50, IC50 and LC50. In studies where even the highest exposure concentration showed no adverse effect on the test organism, this value was included as the Highest Observed No Effect Concentration (HONEC).

### Data evaluation

In most cases, chronic NOEC values, which are needed for the derivation of the PNEC value [25], were not available. Thus each of the ecotoxicological endpoints was transformed by two different assessment factors (AFs) based on the REACH guidance [25]. The first AF is used to extrapolate the observed effect into no effect concentrations. An AF of 10 was used for LC/EC/IC_25–50_, an AF of 2 for LC/EC/IC_10–20_, MIC and LOEC, and an AF of 1 for HONEC and NOEC. The second assessment factor accounts for the extrapolation from short- to long-term effects. Acute studies received higher AFs than studies reporting chronic effects. Long-term studies were assigned an AF of 1, whereas short-term studies received an AF of 10. More information regarding the selection of the assessment factors can be found in a previous study [26].

The collected endpoints were from materials of different morphology (round, oval, spherical, rod-shaped, sheets, needle-shaped, etc.) and different size (up to 400 nm for certain NBMs). Overall, there were 231 data points collected. The results are summarized in Table 1. All data points and their respective assessment factors can be found in Additional file 1: Tables S1–S6. No data was found for PLGA, PLA or PHA. Therefore no hazard assessment could be conducted for these materials. Most ecotoxicological experiments useful for our hazard assessment were conducted on freshwater organisms, while there were only a few studies on soil organisms. No ecotoxicological studies were found for other environmental compartments (i.e. sediment and marine systems). Many studies worked with pathogenic bacteria and were included in a first overall assessment. The reason behind this is that many studies focused on the antimicrobial properties of dissolved chitosan/nano-chitosan instead of their ecological impacts, and thus most of the test organisms were pathogenic bacteria and fungi. A final assessment was performed without these bacteria, designated as “environmental organisms”. By removing these data points, the ecotoxicity for all other environmental organisms are displayed in a more explicit way. Additionally, in studies where it was specially mentioned that the particles were “nanoparticles”, this designation was checked for the reported particles size. If nothing was mentioned, the publications were screened if the work used the dissolved or bulk form of the material and then characterized as “non-nano”. During the preparation of chitosan for exposure, the material is dissolved and therefore the term dissolved was used for non-nano chitosan.

**Table 1:** Summary of the number of ecotoxicological endpoints found for the selected NBMs

NBM	Compartment	# Endpoints	# Species	# Taxonomic groups	# Endpoints on environmental organisms^a^
Nano-chitosan	Freshwater	16	7	3	4
	Soil	0	0	0	0
Non-nano chitosan^b^	Freshwater	138	25	5	5
	Soil	60	8	2	0
Nano-HAP	Freshwater	13	6	3	8
	Soil	1	1	1	0
Non-nano HAP	Freshwater	0	0	0	0
	Soil	0	0	0	0
PAN nanofibers	Freshwater	2	1	1	0
	Soil	1	1	1	0
Non-nano PAN	Freshwater	0	0	0	0
	Soil	0	0	0	0
Nano-PLGA	Freshwater	0	0	0	0
	Soil	0	0	0	0
Non-nano PLGA	Freshwater	0	0	0	0
	Soil	0	0	0	0
Nano-PLA	Freshwater	0	0	0	0
	Soil	0	0	0	0
Non-nano PLA	Freshwater	0	0	0	0
	Soil	0	0	0	0
Nano-PHA	Freshwater	0	0	0	0
	Soil	0	0	0	0
Non-nano PHA	Freshwater	0	0	0	0
	Soil	0	0	0	0

^a^Environmental organism stands for all species except for pathogenic bacteria and fungi

^b^Dissolved

### Species sensitivity distribution modeling

The endpoints collected in Table 1 were converted into PNECs based on two approaches. If only one ecotoxicological endpoint was available for a certain NBM, then an assessment factor of 1000 was applied on the lowest EC50 or, if an EC50 value was not available, on the reported endpoint as suggested by the REACH guideline [25]. If several endpoints were available across multiple species, an SSD was constructed. The ecotoxicological endpoint concentrations were first converted to chronic NOEC values using the assessment factors described above. Then a probabilistic species sensitivity distribution (pSSD) [31] was calculated for every substance, using all the endpoints available. A PNEC value was extracted as the 5th percentile of the pSSDs as recommended by the REACH guidance [25]. With 10,000 simulation runs and one PNEC extracted per run, a PNEC probability distribution was derived.

## Results

### Nanoparticle characterization

**Fig. 1:**
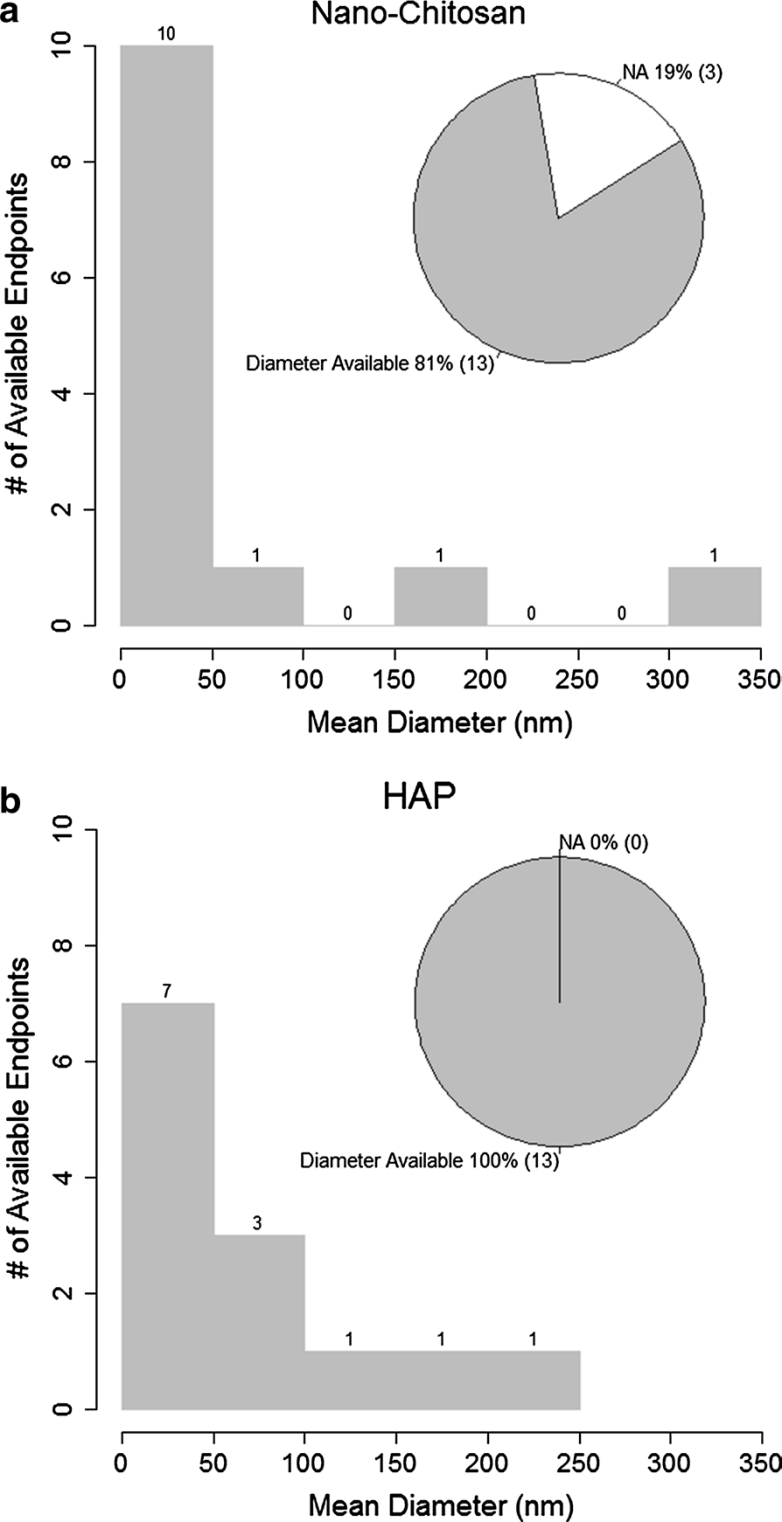
Summary of size distributions of nano-chitosan and HAP used in freshwater ecotoxicity studies. “NA”: size not reported in the study

The nanoparticle size distributions of nano-chitosan and HAP used in freshwater studies is shown in Fig. 1. The figure also shows the percentage of data points for which the particle diameter was reported. The mean diameters ranged from 0 to 350 nm for nano-chitosan and from 0 to 250 nm for HAP nanoparticles. For nano-chitosan, the diameter was available for 81% of the data points whereas for HAP, the mean diameter was available for all the data points. The shapes of the nanoparticles varied greatly for different materials in different studies (Additional file 1: Table S7). Most chitosan nanoparticles are round or oval shaped; some are agglomerated in clusters. Most HAP nanoparticles are rod shaped while PAN is mostly in the shape of nanofibers and loaded with other materials.

### Probabilistic species sensitivity distributions (pSSDs)

**Fig. 2:**
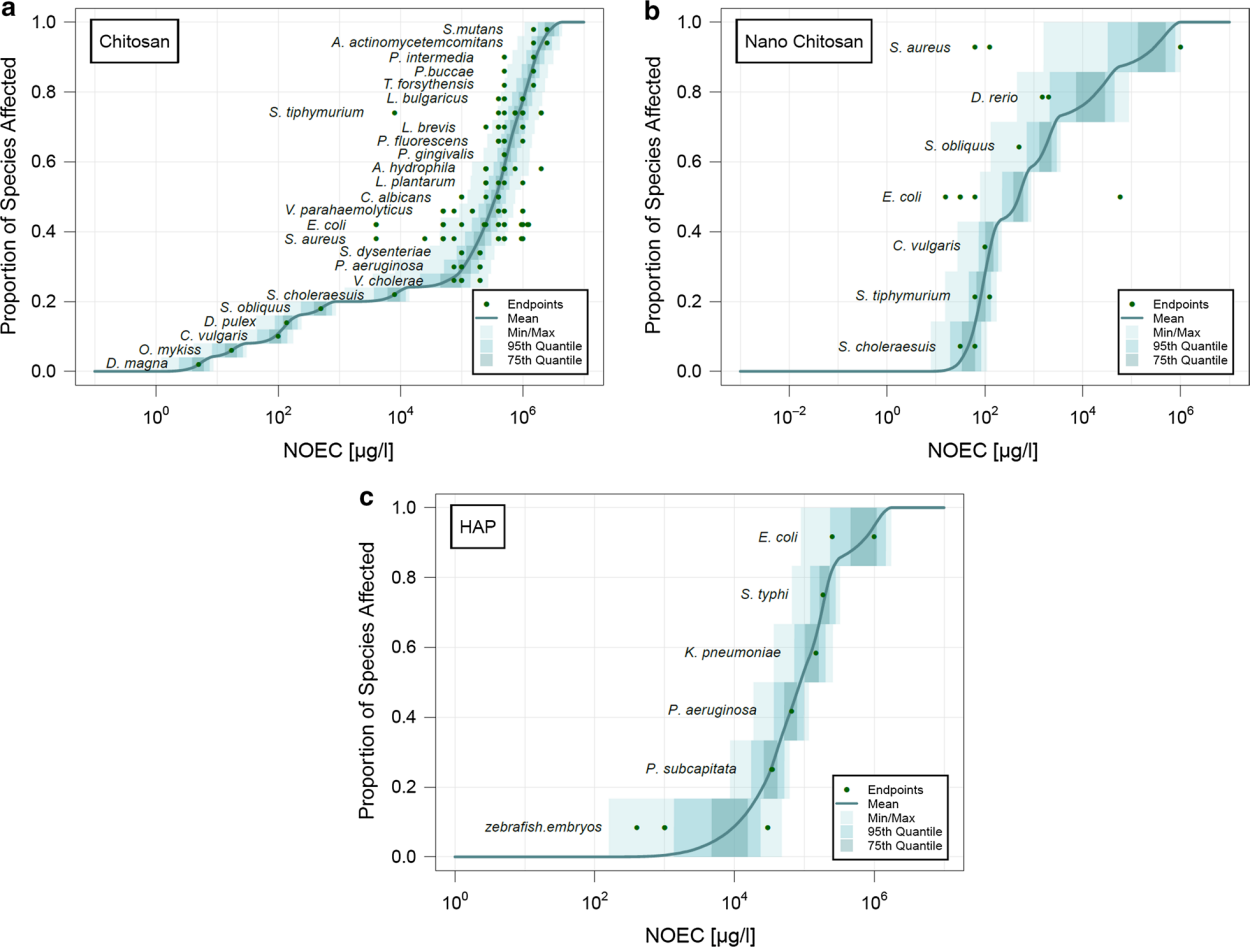
Probabilistic species sensitivity distributions (pSSDs) of chitosan, chitosan nanoparticles, and HAP nanoparticles in freshwater

The pSSDs for chitosan, nano-chitosan, and HAP in freshwater are shown in Fig. 2. Regarding chitosan in freshwater, the nano and dissolved form are shown. The shadings show the maximum, minimum, 95th and 75th quantiles; the line shows the mean of all the individual pSSDs. The individual NOECs derived from the different endpoints were grouped together by species and are shown as dots. For many species, the range of individual NOEC values spans many orders of magnitude. For example, the different NOEC values for *E. coli*, *S. aureus* and *S. typhi* for chitosan in freshwater from different studies range over three orders of magnitude (see Fig. 2a). This can be attributed to a number of uncertainties, such as different nanoparticles properties, different experimental conditions, etc. Some species were tested regarding their toxicity to several NBMs. For example, the toxicity of *E. coli* was tested for chitosan, nano-chitosan and HAP. While *E. coli* is the least sensitive species regarding HAP, it is around average for chitosan and nano-chitosan.

**Fig. 3:**
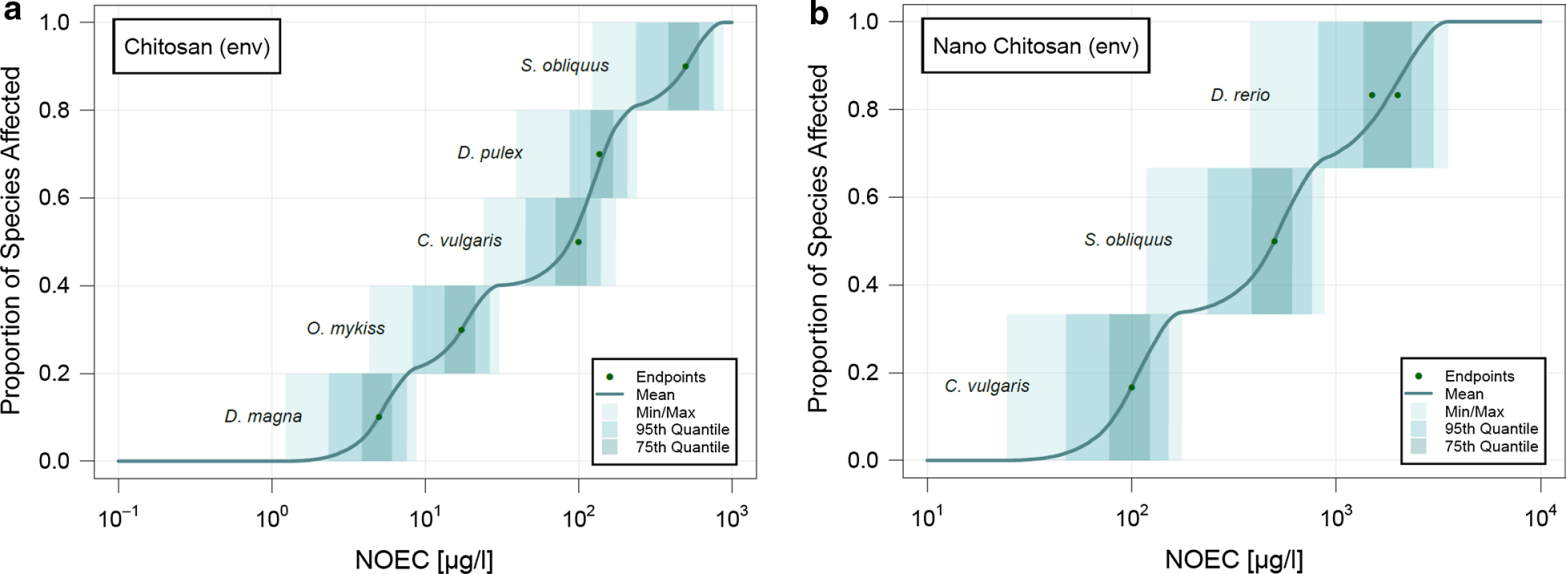
Probabilistic species sensitivity distributions (pSSDs) of chitosan and chitosan nanoparticles in freshwater by removing all pathogenic bacteria and fungi in the collected data

The final evaluation was performed where all pathogenic bacteria and fungi were removed from the original pSSD for dissolved and nano-chitosan in freshwater. These pSSDs are shown in Fig. 3. For chitosan, the five most sensitive species are the environmental species, while all the other species are pathogenic bacteria or fungi. Therefore, pathogenic bacteria and fungi are less sensitive to chitosan than the environmental species. The environmental species for nano-chitosan on the other hand are located somewhere in the middle of the pSSD.

**Fig. 4:**
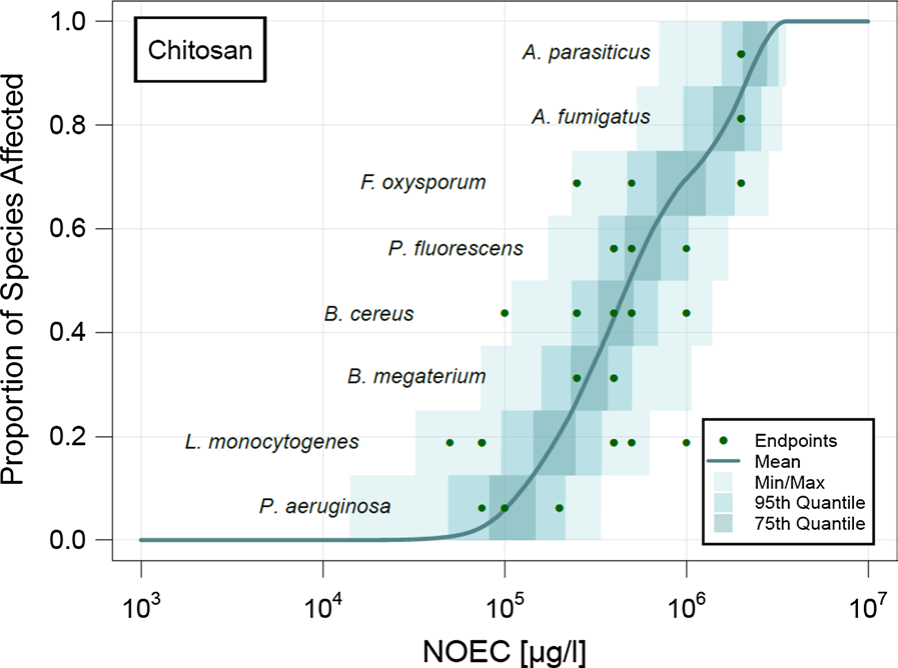
Probabilistic species sensitivity distribution (pSSD) of chitosan in soil

For soil, only enough data to calculate a pSSD was a found for dissolved chitosan, which is shown in Fig. 4. No data was found for the nano form. For HAP and PAN, only one ecotoxicological endpoint was available for each of these substances and therefore only a PNEC but no pSSD could be calculated.

**Fig. 5:**
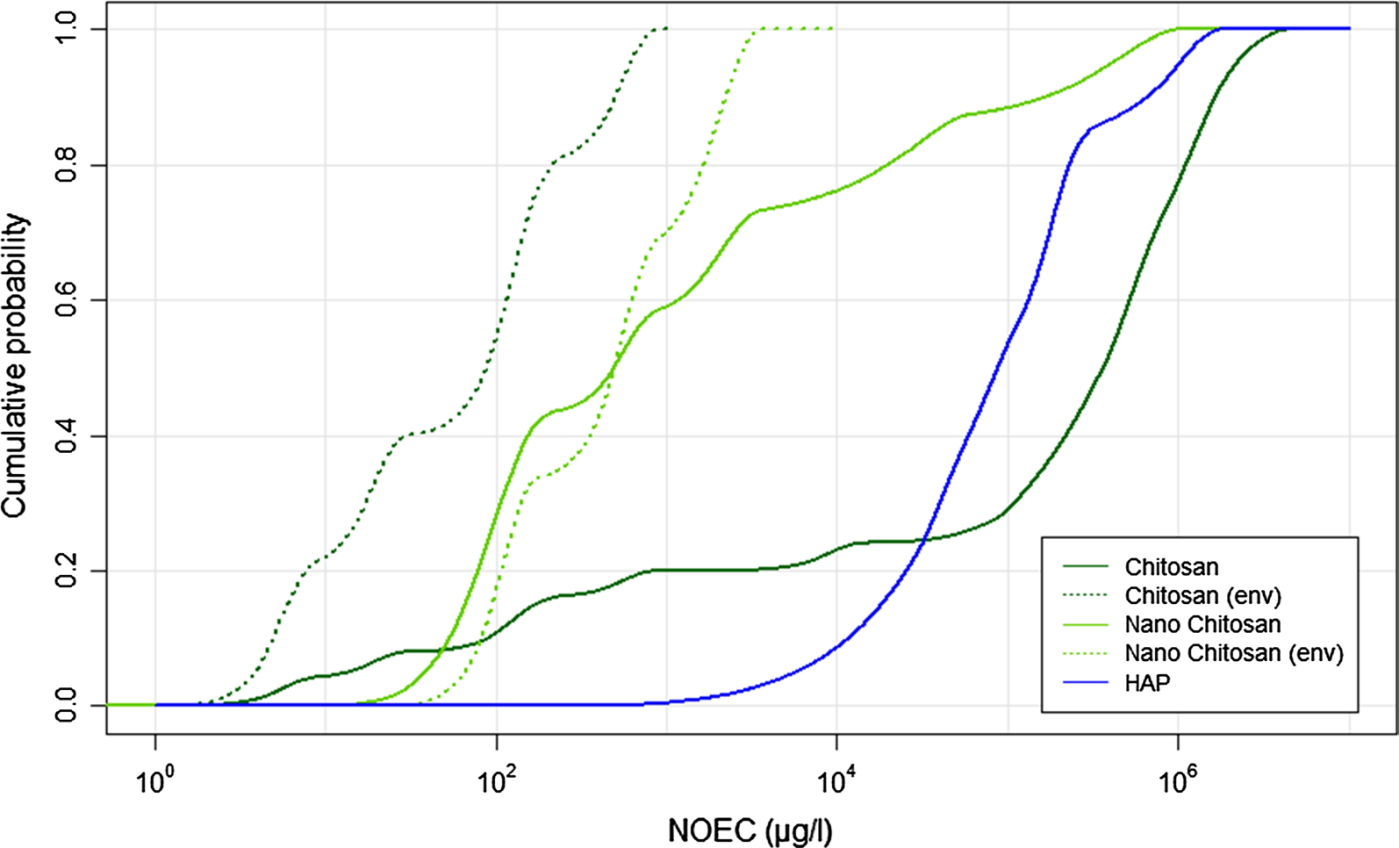
Cumulative probabilistic species sensitivity distributions (pSSDs) of chitosans and HAP in freshwater. For chitosan, curves are shown for all data and for pathogenic bacteria removed (labelled “env”)

In order to allow a comparison between the different materials, the mean pSSDs in freshwater of all compounds are plotted together in Fig. 5. As it was only possible to calculate a pSSD in soil for chitosan, no such comparison is shown for the soil compartment. The figure shows that chitosan has a very wide distribution of toxicity data, ranging over six orders of magnitude from 10^0^ to 10^6^ μg/l. Chitosan and nano-chitosan on the other hand have a much steeper pSSD with toxicity data only spread over three orders of magnitude.

### Predicted no-effect concentration (PNEC) distribution

The PNEC distribution for each NBM was derived by calculating the 5th percentile of each single pSSD. The distribution is different for each nanomaterial and is shown for freshwater in Additional file 1: Figure S1. The median, mean and mode of the PNEC distributions were calculated. They are shown for each NBM including their nanoform (if available) in Table 2 for freshwater and Table 3 for soil. The minimum, maximum and the 25th and 75th percentiles are also listed. No distribution can be shown for PAN in freshwater as well as PAN and HAP in soil, as only one endpoint was available in each case. From Tables 2 and 3 it can be seen that the PNEC for freshwater and soil increases from most sensitive to least sensitive in the following order:

**Table 2:** Median, mean, mode, minimum, maximum, 25th and 75th quantiles from predicted no-effect concentration (PNEC) distributions in freshwater

[μg/l]	Chitosan^a^	Nano-Chitosan^a^	HAP	PAN
Median	5	99	9.4*10^3^	NA^b^
Mean	5	100	1.1*10^4^	3.0*10^6^
Mode	5	97	2.7*10^3^	NA
Min	1	25	120	NA
25%	4	78	4.4*10^3^	NA
75%	6	120	1.5*10^4^	NA
Max	8	180	5.0*10^4^	NA

All values in µg/l (The values for chitosan and nano-chitosan using all available endpoints can be found in Additional file 1: Table S9)

^a^PNEC values only considering environmental species

^b^No distribution as only one endpoint was found

**Table 3:** Median, mean, mode, minimum, maximum, 25th and 75th quantile from predicted no-effect concentration (PNEC) distributions in soil

[mg/kg]	Chitosan	HAP	PAN
Median	110	NA^a^	NA^a^
Mean	119	0.3	33
Mode	97	NA	NA
Min	12	NA	NA
25%	83	NA	NA
75%	148	NA	NA
Max	335	NA	NA

All values in mg/kg

^a^No distribution as only one endpoint was found

- Freshwater: Chitosan < Nano-Chitosan ≪ HAP ≪ PAN
- Soil: HAP < PAN < Chitosan

These orders indicate that PAN nanofibers have the highest predicted no-effect concentration in freshwater and the second highest in soil and are therefore expected to be the least toxic of the investigated NBMs. It is also interesting to note that HAP has a high PNEC in freshwater but the lowest PNEC among the investigated materials in soil. This could imply that the toxicity of a material might also be dependent on the media they are in.

## Discussion

The availability of ecotoxicological data differs significantly between the investigated NBMs. For chitosan many ecotoxicological studies were available whereas for PLA, PHA and PLGA none were found. There are several reasons for the lack of data for some NBMs in certain environmental compartments. For PAN, some toxicity studies were found but most of them focused on in vitro cytotoxicity and on animal tests (rats and mice) in the context of human risk assessment. Additionally, ecological endpoints for a number of studies on nano-PAN membranes and PAN nanofibers were not available because the exact concentrations used were not mentioned in the study. While there are sufficient data for chitosan and HAP, their antimicrobial (antibacterial/fungicidal) properties were of more concern in many studies. This is caused by the antimicrobial application of the studied materials (e.g. wound dressings, tissue scaffolds and bone engineering). As a result, the majority of effect studies focused on pathogenic microorganisms and should not be used directly for an ecotoxicological evaluation.

Due to their biodegradability [32, 33], the amounts of residues from polymeric NBMs released to the ecosystem are expected to be substantially reduced. Most toxicological studies of these materials showed therefore more concern for human risks (in vivo) than for ecological hazard. For example, PLGA is regarded as one of the most successfully used biodegradable nanosystems for the development of nanomedicines as it undergoes hydrolysis in the body to produce the biodegradable metabolites lactic acid and glycolic acid which are easily metabolized by the body. Therefore, PLGA is not expected to be present in the environment at elevated concentrations and ecotoxicological effects studies on this material are rarely conducted.

Another reason for the lack of data is that in some studies, the NBM, particularly PAN and PLGA nanofibers/nanoparticles, was not the center of the experiment but the toxicity of the active substance. Therefore only the control or blank was relevant for our evaluation. Special care was taken to discard experiments for HAP that used metal-doped hydroxyapatite nanoparticles as this increases the toxicity to the test organisms, which leads to an overestimation of the toxicity. There is much less data available for the soil system than for freshwater. One reason for this shortage of data is that several ecotoxicological studies could not be considered because soil organisms were studied in aqueous suspensions and these testing conditions were deemed inappropriate for risk assessment purposes [27]. Furthermore, it should not be forgotten that journals often discard studies that show no effect on the studied organism [34]. This triggers possible bias or overestimation of the environmental effects (i.e. ecotoxicity), especially for the relatively less toxic PAN nanofibers and PLGA nanoparticles.

For chitosan, which was found to be the most toxic of the investigated NBMs in freshwater, a lot of endpoints were found for several different species. For PAN on the other hand, only one endpoint was found showing almost no toxicity. It is understandable that a material with expected low toxicity is less studied than a material which is fairly toxic.

Additionally, attention needs to be taken when using the PNEC calculated from only one available endpoint. For HAP in freshwater, two endpoints were available: an EC50 and a HONEC for *E. coli*. The EC50 value was taken and divided by the assessment factor to derive the PNEC. For HAP and PAN in soil, only one endpoint each was found: a MIC from *K. pneumoniae* for HAP and a HONEC from *B. cereus* for PAN. In the absence of better data, these values represent a first indication of toxicity for these materials.

The REACH guidelines state that confidence can be associated with a PNEC derived by statistical extrapolation if the database contains at least 10 NOECs (preferably more than 15) for different species covering at least eight taxonomic groups [25]. In this study, more than 10 NOECs were only found for nano-chitosan in freshwater (16 endpoints), dissolved chitosan in freshwater (138) and soil (60), and nano-HAP in freshwater (13). For all the evaluated NBMs, data points were obtained for less than eight taxonomic groups. Dissolved chitosan in freshwater covered the highest number of taxonomic groups in this study with data from five taxonomic groups. Although the data are not good enough for strict regulatory risk assessment, they provide a first analysis of the available ecotoxicity data.

One aim of the work was to evaluate if there is any nano-specific toxicity of NBMs. This could only be evaluated for chitosan where data were available both for dissolved and nano-chitosan. Conventionally, nanoparticles are defined as particles between 1 and 100 nm in size [35], however the term “nanoparticles” is also used in the literature for NBMs whose sizes are larger than 100 nm. This contradiction should be noticed when making policies or guidelines for such “nanoparticles”. Moreover, the number of available information on nanoparticle size distributions was very limited, in some studies even missing completely (as shown in Fig. 1). The available data suggest that the nano-form of chitosan is less toxic than the dissolved form. This is similar to data for other nanoparticles that can dissolve, e.g. nano-Ag, nano-CuO and nano-ZnO, where also the dissolved form was found to be much more toxic than the nano-form [36].

**Fig. 6:**
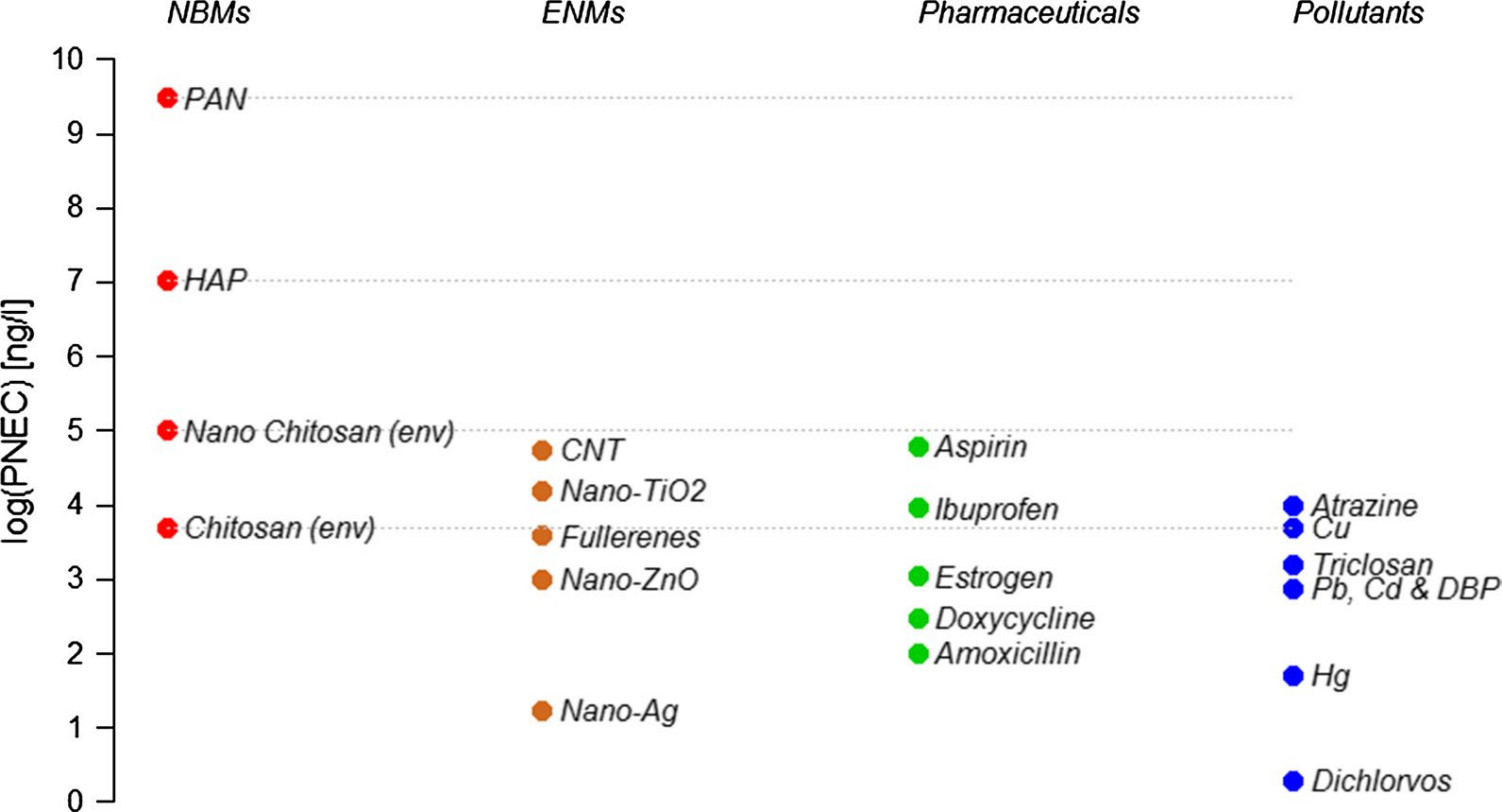
Predicted no-effect concentrations (PNEC) of nanobiomaterials (NBMs), engineered nanomaterials (ENMs), pharmaceuticals and some pollutants in freshwater. For references see Additional file 1: Table S8

Figure 6 compares the PNEC values in the freshwater compartment for the studied NBMs (red dots) and for several other common pollutants: engineered nanomaterials (brown), pharmaceuticals (green) and other pollutants such as metals and pesticides (blue). If available, the PNEC using only environmental species was used. The details and corresponding references are shown in Additional file 1: Table S8. Generally, chitosan has a relatively high toxicity in freshwater, comparable to other ENMs, while PAN and HAP can be treated as almost non-toxic. Chitosan has a similar ecotoxicity to many other pharmaceuticals which have been studied for their effects on organisms. The nano-form of chitosan is, however, less toxic than common ENMs, the antibiotics estrogen, doxycycline and amoxicillin, the heavy metals Cu, Pb, Cd and Hg and the organic pollutants triclosan, dibutylphtalate (DBP) and dichlorvos. So in summary, based on the available data for the studied NBMs there is an indication that nano-chitosan might to be of highest concern for the environment, while PAN and HAP seem to not represent significant toxicity. It is important to note here that we can only present an evaluation of the environmental hazard and thus cannot make any claims about environmental risks. In order to perform an environmental risk assessment, we would also need information on the environmental exposure of the considered NBMs, which is currently not available. The only risk assessment of a nanomaterial used in a medical context published so far is for nano-Au [30]. Future studies need to provide more information on environmental exposure of NBMs so that full risk assessments for more materials can be performed.

## Additional file

**Additional file 1.** Additional tables and figure.
