# Supplementary material for: Environmental hazard assessment for polymeric and inorganic nanobiomaterials used in drug delivery
Source: J Nanobiotechnology. 2019 Apr 16;17:56. doi: 10.1186/s12951-019-0489-8 (PMC6466702; doi:10.1186/s12951-019-0489-8)
Supplement: Supplementary file 1 — Additional file 1. Additional tables and figure. [file 12951_2019_489_MOESM1_ESM.pdf]

Environmental Hazard Assessment for Polymeric and Inorganic Nanobiomaterials used in Drug Delivery

Marina Hauser, Guangyu Li, Bernd Nowack*

Empa, Swiss Federal Laboratories for Materials Science and Technology, Lerchenfeldstrasse 5, 9014 St. Gallen, Switzerland

*Address correspondence to nowack@empa.ch

Additional file 1

9 Tables

1 Figure

Table S1: Data for freshwater toxicity of chitosan

Reference	Taxonomy	Test organisms	Ecotoxical endpoint	Concentration (µg/L) (nano-sized)	Exposure time (h)	AF-time	AF-NOEC	species sensitivity (µg/L) = concentration/AF
Costa et al (2012)	bacterium	P. gingivalis	MIC	1,000,000	72	1	2	500,000
Costa et al (2012)	bacterium	P. gingivalis	MIC	1,000,000	72	1	2	500,000
Costa et al (2012)	bacterium	T. forsythensis	MIC	1,000,000	72	1	2	500,000
Costa et al (2012)	bacterium	T. forsythensis	MIC	3,000,000	72	1	2	1,500,000
Costa et al (2012)	bacterium	P.buccae	MIC	3,000,000	72	1	2	1,500,000
Costa et al (2012)	bacterium	P.buccae	MIC	1,000,000	72	1	2	500,000
Costa et al (2012)	bacterium	A. actinomycetemcomitans	MIC	5,000,000	72	1	2	2,500,000
Costa et al (2012)	bacterium	A. actinomycetemcomitans	MIC	3,000,000	72	1	2	1,500,000
Costa et al (2012)	bacterium	P. intermedia	MIC	1,000,000	72	1	2	500,000
Costa et al (2012)	bacterium	P. intermedia	MIC	3,000,000	72	1	2	1,500,000
Tsai et al (2002)	bacterium	E. coli	MIC	100,000	48	1	2	50,000
Tsai et al (2002)	bacterium	E. coli	MIC	100,000	48	1	2	50,000
Tsai et al (2002)	bacterium	E. coli	MIC	100,000	48	1	2	50,000
Tsai et al (2002)	bacterium	E. coli	MIC	100,000	48	1	2	50,000
Tsai et al (2002)	bacterium	E. coli	MIC	500,000	48	1	2	250,000
Tsai et al (2002)	bacterium	E. coli	MIC	200,000	48	1	2	100,000
Du et al (2008)	bacterium	E. coli	MIC	468,000	24	1	2	234,000
No et al (2002)	bacterium	E. coli	MIC	1,000,000	72	1	2	500,000
No et al (2002)	bacterium	E. coli	HONEC	1,000,000	72	1	1	1,000,000
No et al (2002)	bacterium	E. coli	MIC	800,000	72	1	2	400,000
No et al (2002)	bacterium	E. coli	MIC	800,000	72	1	2	400,000
No et al (2002)	bacterium	E. coli	MIC	1,000,000	72	1	2	500,000
No et al (2002)	bacterium	E. coli	HONEC	1,000,000	72	1	1	1,000,000
Du et al (2008)	bacterium	E. coli	MIC	**117,000**	24	1	2	58,500
Fernandes et al (2008)	bacterium	E. coli	MIC	1,900,000	24	1	2	950,000
Fernandes et al (2008)	bacterium	E. coli	MIC	2,400,000	24	1	2	1,200,000
Fernandes et al (2008)	bacterium	E. coli	MIC	2,500,000	24	1	2	1,250,000
Qi et al (2004)	bacterium	E. coli	MIC	**125**	24	1	2	62.50
Qi et al (2004)	bacterium	E. coli	MIC	8,000	24	1	2	4,000
Qi et al (2004)	bacterium	E. coli	MIC	**62.50**	24	1	2	31.250
Qi et al (2004)	bacterium	E. coli	MIC	**62.50**	24	1	2	31.250
Qi et al (2004)	bacterium	E. coli	MIC	8,000	24	1	2	4,000
Qi et al (2004)	bacterium	E. coli	MIC	**31.25**	24	1	2	15.6250
Tsai et al (2002)	bacterium	P. aeruginosa	MIC	200,000	48	1	2	100,000
Tsai et al (2002)	bacterium	P. aeruginosa	MIC	150,000	48	1	2	75,000
Tsai et al (2002)	bacterium	P. aeruginosa	HONEC	200,000	48	1	1	200,000
Tsai et al (2002)	bacterium	P. aeruginosa	MIC	200,000	48	1	2	100,000
Tsai et al (2002)	bacterium	P. aeruginosa	HONEC	200,000	48	1	1	200,000
Tsai et al (2002)	bacterium	P. aeruginosa	HONEC	200,000	48	1	1	200,000
No et al (2002)	bacterium	P. fluorescens	MIC	1,000,000	72	1	2	500,000
No et al (2002)	bacterium	P. fluorescens	HONEC	1,000,000	72	1	1	1,000,000
No et al (2002)	bacterium	P. fluorescens	MIC	800,000	72	1	2	400,000
No et al (2002)	bacterium	P. fluorescens	MIC	800,000	72	1	2	400,000
No et al (2002)	bacterium	P. fluorescens	MIC	800,000	72	1	2	400,000
No et al (2002)	bacterium	P. fluorescens	MIC	1,000,000	72	1	2	500,000
Qi et al (2004)	bacterium	S. choleraesuis	MIC	**125**	24	1	2	62.50
Qi et al (2004)	bacterium	S. choleraesuis	MIC	16,000	24	1	2	8,000
Qi et al (2004)	bacterium	S. choleraesuis	MIC	**62.50**	24	1	2	31.250
No et al (2002)	bacterium	S. tiphymurium	HONEC	1,000,000	72	1	1	1,000,000
No et al (2002)	bacterium	S. tiphymurium	HONEC	1,000,000	72	1	1	1,000,000
No et al (2002)	bacterium	S. tiphymurium	HONEC	1,000,000	72	1	1	1,000,000
No et al (2002)	bacterium	S. tiphymurium	MIC	800,000	72	1	2	400,000
No et al (2002)	bacterium	S. tiphymurium	MIC	1,000,000	72	1	2	500,000
No et al (2002)	bacterium	S. tiphymurium	HONEC	1,000,000	72	1	1	1,000,000
Tsai et al (2002)	bacterium	S. tiphymurium	MIC	1,500,000	48	1	2	750,000
Tsai et al (2002)	bacterium	S. tiphymurium	MIC	1,500,000	48	1	2	750,000
Tsai et al (2002)	bacterium	S. tiphymurium	MIC	1,500,000	48	1	2	750,000
Tsai et al (2002)	bacterium	S. tiphymurium	MIC	1,500,000	48	1	2	750,000

Table S1: Data for freshwater toxicity of chitosan (cont.)

Reference	Taxonomy	Test organisms	Ecotoxical endpoint	Concentration (µg/L) (nano-sized)	Exposure time (h)	AF-time	AF-NOEC	species sensitivity (µg/L) = concentration/AF
Tsai et al (2002)	bacterium	S. tiphymurium	HONEC	2,000,000	48	1	1	2,000,000
Tsai et al (2002)	bacterium	S. tiphymurium	HONEC	2,000,000	48	1	1	2,000,000
Qi et al (2004)	bacterium	S. tiphymurium	MIC	**125**	24	1	2	63
Qi et al (2004)	bacterium	S. tiphymurium	MIC	16,000	24	1	2	8,000
Qi et al (2004)	bacterium	S. tiphymurium	MIC	**250**	24	1	2	125.00
Tsai et al (2002)	bacterium	S. dysenteriae	MIC	500,000	48	1	2	250,000
Tsai et al (2002)	bacterium	A. hydrophila	MIC	500,000	48	1	2	250,000
Tsai et al (2002)	bacterium	A. hydrophila	MIC	500,000	48	1	2	250,000
Tsai et al (2002)	bacterium	A. hydrophila	MIC	500,000	48	1	2	250,000
Tsai et al (2002)	bacterium	A. hydrophila	HONEC	500,000	48	1	1	500,000
Tsai et al (2002)	bacterium	A. hydrophila	HONEC	500,000	48	1	1	500,000
Tsai et al (2002)	bacterium	A. hydrophila	MIC	500,000	48	1	2	250,000
Tsai et al (2002)	bacterium	A. hydrophila	MIC	1,000,000	48	1	2	500,000
Tsai et al (2002)	bacterium	A. hydrophila	MIC	1,000,000	48	1	2	500,000
Tsai et al (2002)	bacterium	A. hydrophila	MIC	1,000,000	48	1	2	500,000
Tsai et al (2002)	bacterium	A. hydrophila	MIC	1,500,000	48	1	2	750,000
Tsai et al (2002)	bacterium	A. hydrophila	HONEC	2,000,000	48	1	1	2,000,000
Tsai et al (2002)	bacterium	S. dysenteriae	MIC	200,000	48	1	2	100,000
Tsai et al (2002)	bacterium	S. dysenteriae	MIC	200,000	48	1	2	100,000
Tsai et al (2002)	bacterium	S. dysenteriae	HONEC	200,000	48	1	1	200,000
Tsai et al (2002)	bacterium	S. dysenteriae	HONEC	200,000	48	1	1	200,000
Tsai et al (2002)	bacterium	S. dysenteriae	HONEC	200,000	48	1	1	200,000
Tsai et al (2002)	bacterium	S. dysenteriae	HONEC	200,000	48	1	1	200,000
Tsai et al (2002)	bacterium	V. cholerae	MIC	150,000	48	1	2	75,000
Tsai et al (2002)	bacterium	V. cholerae	MIC	200,000	48	1	2	100,000
Tsai et al (2002)	bacterium	V. cholerae	MIC	200,000	48	1	2	100,000
Tsai et al (2002)	bacterium	V. cholerae	MIC	200,000	48	1	2	100,000
Tsai et al (2002)	bacterium	V. cholerae	HONEC	200,000	48	1	1	200,000
Tsai et al (2002)	bacterium	V. cholerae	HONEC	200,000	48	1	1	200,000
Tsai et al (2002)	bacterium	V. parahaemolyticus	MIC	100,000	48	1	2	50,000
Tsai et al (2002)	bacterium	V. parahaemolyticus	MIC	100,000	48	1	2	50,000
Tsai et al (2002)	bacterium	V. parahaemolyticus	MIC	150,000	48	1	2	75,000
Tsai et al (2002)	bacterium	V. parahaemolyticus	MIC	100,000	48	1	2	50,000
Tsai et al (2002)	bacterium	V. parahaemolyticus	HONEC	150,000	48	1	1	150,000
Tsai et al (2002)	bacterium	V. parahaemolyticus	HONEC	150,000	48	1	1	150,000
No et al (2002)	bacterium	V. parahaemolyticus	MIC	1,000,000	72	1	2	500,000
No et al (2002)	bacterium	V. parahaemolyticus	HONEC	1,000,000	72	1	1	1,000,000
No et al (2002)	bacterium	V. parahaemolyticus	MIC	800,000	72	1	2	400,000
No et al (2002)	bacterium	V. parahaemolyticus	MIC	800,000	72	1	2	400,000
No et al (2002)	bacterium	V. parahaemolyticus	MIC	1,000,000	72	1	2	500,000
No et al (2002)	bacterium	V. parahaemolyticus	HONEC	1,000,000	72	1	1	1,000,000
Costa et al (2012)	bacterium	S. mutans	MIC	3,000,000	72	1	2	1,500,000
Costa et al (2012)	bacterium	S. mutans	MIC	5,000,000	72	1	2	2,500,000
Tsai et al (2002)	bacterium	S. aureus	MIC	100,000	48	1	2	50,000
Tsai et al (2002)	bacterium	S. aureus	MIC	50,000	48	1	2	25,000
Tsai et al (2002)	bacterium	S. aureus	MIC	100,000	48	1	2	50,000
Tsai et al (2002)	bacterium	S. aureus	MIC	100,000	48	1	2	50,000
Tsai et al (2002)	bacterium	S. aureus	MIC	100,000	48	1	2	50,000
Tsai et al (2002)	bacterium	S. aureus	MIC	150,000	48	1	2	75,000
No et al (2002)	bacterium	S. aureus	MIC	1,000,000	48	1	2	500,000
No et al (2002)	bacterium	S. aureus	HONEC	1,000,000	48	1	1	1,000,000
No et al (2002)	bacterium	S. aureus	MIC	800,000	48	1	2	400,000
No et al (2002)	bacterium	S. aureus	MIC	800,000	48	1	2	400,000
No et al (2002)	bacterium	S. aureus	MIC	800,000	48	1	2	400,000
No et al (2002)	bacterium	S. aureus	HONEC	1,000,000	48	1	1	1,000,000
Fernandes et al (2008)	bacterium	S. aureus	MIC	1,900,000	24	1	2	950,000
Fernandes et al (2008)	bacterium	S. aureus	MIC	1,000,000	24	1	2	500,000
Fernandes et al (2008)	bacterium	S. aureus	MIC	1,000,000	24	1	2	500,000
Qi et al (2004)	bacterium	S. aureus	MIC	**250**	24	1	2	125.0
Qi et al (2004)	bacterium	S. aureus	MIC	**125**	24	1	2	62.5
Qi et al (2004)	bacterium	S. aureus	MIC	8,000	24	1	2	4,000
Sadeghi et al (2008)	bacterium	S. aureus	MIC	1,000,000	24	1	2	500,000

Table S1: Data for freshwater toxicity of chitosan (cont.)

Reference	Taxonomy	Test organisms	Ecotoxical endpoint	Concentration (μg/L) (nano-sized)	Exposure time (h)	AF-time	AF-NOEC	species sensitivity (μg/L) = concentration/AF
Sadeghi et al (2008)	bacterium	S. aureus	MIC	**2,000,000**	24	1	2	1,000,000
No et al (2002)	bacterium	L. plantarum	HONEC	1,000,000	72	1	1	1,000,000
No et al (2002)	bacterium	L. plantarum	MIC	800,000	72	1	2	400,000
No et al (2002)	bacterium	L. plantarum	MIC	500,000	72	1	2	250,000
No et al (2002)	bacterium	L. plantarum	MIC	1,000,000	72	1	2	500,000
No et al (2002)	bacterium	L. plantarum	MIC	500,000	72	1	2	250,000
No et al (2002)	bacterium	L. plantarum	MIC	500,000	72	1	2	250,000
No et al (2002)	bacterium	L. bulgaricus	HONEC	1,000,000	72	1	1	1,000,000
No et al (2002)	bacterium	L. bulgaricus	MIC	800,000	72	1	2	400,000
No et al (2002)	bacterium	L. bulgaricus	HONEC	1,000,000	72	1	1	1,000,000
No et al (2002)	bacterium	L. bulgaricus	HONEC	1,000,000	72	1	1	1,000,000
No et al (2002)	bacterium	L. bulgaricus	MIC	1,000,000	72	1	2	500,000
No et al (2002)	bacterium	L. bulgaricus	MIC	1,000,000	72	1	2	500,000
No et al (2002)	bacterium	L. brevis	MIC	800,000	72	1	2	400,000
No et al (2002)	bacterium	L. brevis	MIC	500,000	72	1	2	250,000
No et al (2002)	bacterium	L. brevis	HONEC	1,000,000	72	1	1	1,000,000
No et al (2002)	bacterium	L. brevis	MIC	1,000,000	72	1	2	500,000
No et al (2002)	bacterium	L. brevis	HONEC	1,000,000	72	1	1	1,000,000
No et al (2002)	bacterium	L. brevis	MIC	800,000	72	1	2	400,000
Tsai et al (2002)	fungi	C. albicans	MIC	200,000	48	1	2	100,000
Tsai et al (2002)	fungi	C. albicans	MIC	200,000	48	1	2	100,000
Tsai et al (2002)	fungi	C. albicans	MIC	500,000	48	1	2	250,000
Tsai et al (2002)	fungi	C. albicans	MIC	800,000	48	1	2	400,000
Tsai et al (2002)	fungi	C. albicans	MIC	800,000	48	1	2	400,000
Tsai et al (2002)	fungi	C. albicans	MIC	800,000	48	1	2	400,000
Hu et al (2011)	fish	D. rerio	LOEC	**40,000**	96	10	2	2,000
Hu et al (2011)	fish	D. rerio	LOEC	**30,000**	96	10	2	1,500
Sigma-Aldrich (2016)	fish	O. mykiss	LC50	1,730	96	10	10	17.3
Wen et al (2010)	algae	C. vulgaris	EC27	1,000	168	1	10	100
Wen et al (2010)	algae	C. vulgaris	EC25	**1,000**	168	1	10	100
Wen et al (2010)	algae	S. obliquus	EC15	1,000	168	1	2	500
Wen et al (2010)	algae	S. obliquus	EC17	**1,000**	168	1	2	500
Rizzo et al (2008)	invertebrate	D. magna	EC40	500	24	10	10	5.00
Sigma-Aldrich (2016)	invertebrate	D. pulex	EC50	13,690	48	10	10	136.9

Table S2: Data for freshwater toxicity of HAP

Reference	Taxonomy	Test organisms	Ecotoxical endpoint	Concentration (μg/L)	Exposure time (h)	AF-time	AF-NOEC	species sensitivity (μg/L) = concentratioNAF
Zhao et al (2013)	fish	zebrafish embryos	HONEC	300,000	80	10	1	30,000
Zhao et al (2013)	fish	zebrafish embryos	HONEC	300,000	80	10	1	30,000
Pujari-P. et al (2017)	fish	zebrafish embryos	EC50	100,000	120	10	10	1,000
Pujari-P. et al (2017)	fish	zebrafish embryos	EC50	100,000	72	10	10	1,000
Pujari-P. et al (2017)	fish	zebrafish embryos	EC50	100,000	72	10	10	1,000
Pujari-P. et al (2017)	fish	zebrafish embryos	EC50	40,000	24	10	10	400
Li et al (2010)	bacterium	E.coli	LC50	10,000,000	24	1	10	1,000,000
Baskar et al (2016)	bacterium	E.coli	MIC	500,000	24	1	2	250,000
Baskar et al (2016)	bacterium	P. aeruginosa	MIC	131,700	24	1	2	65,850
Baskar et al (2016)	bacterium	K. pneumoniae	MIC	292,800	24	1	2	146,400
Baskar et al (2016)	bacterium	S. typhi	MIC	370,700	24	1	2	185,350
Pereira et al (2017)	algae	P. subcapitata	IC50	340,000	72	1	10	34,000
Pereira et al (2017)	algae	P. subcapitata	IC50	350,000	72	1	10	35,000

Table S3: Data for freshwater toxicity of PAN

Reference	Taxonomy	Test organisms	Ecotoxical endpoint	Concentration (μg/L)	Exposure time (h)	AF-time	AF-NOEC	species sensitivity (μg/L) = concentratioNAF
He et al 2016	bacterium	E.coli	EC50	3,000,000,000	24	1	10	300,000,000
Shi et al 2011	bacterium	E.coli	HONEC	32,500,000	18	10	1	3,250,000

Table S4: Data for soil toxicity of chitosan

Reference	Taxonomy	Test organisms	Ecotoxical endpoint	Concentration (μg/kg)	Exposure time (h)	AF-time	AF-NOEC	species sensitivity (μg/L) = concentratioNAF
Tsai et al (2002)	bacterium	P. aeruginosa	MIC	200,000	48	1	2	100,000
Tsai et al (2002)	bacterium	P. aeruginosa	MIC	150,000	48	1	2	75,000
Tsai et al (2002)	bacterium	P. aeruginosa	HONEC	200,000	48	1	1	200,000
Tsai et al (2002)	bacterium	P. aeruginosa	MIC	200,000	48	1	2	100,000
Tsai et al (2002)	bacterium	P. aeruginosa	HONEC	200,000	48	1	1	200,000
Tsai et al (2002)	bacterium	P. aeruginosa	HONEC	200,000	48	1	1	200,000
No et al (2002)	bacterium	P. aeruginosa	MIC	1,000,000	72	1	2	500,000
No et al (2002)	bacterium	P. aeruginosa	HONEC	1,000,000	72	1	1	1,000,000
No et al (2002)	bacterium	P. aeruginosa	MIC	800,000	72	1	2	400,000
No et al (2002)	bacterium	P. aeruginosa	MIC	800,000	72	1	2	400,000
No et al (2002)	bacterium	P. aeruginosa	MIC	800,000	72	1	2	400,000
No et al (2002)	bacterium	P. aeruginosa	MIC	1,000,000	72	1	2	500,000
No et al (2002)	bacterium	B. cereus	MIC	800,000	72	1	2	400,000
No et al (2002)	bacterium	B. cereus	HONEC	1,000,000	72	1	1	1,000,000
No et al (2002)	bacterium	B. cereus	MIC	800,000	72	1	2	400,000
No et al (2002)	bacterium	B. cereus	MIC	500,000	72	1	2	250,000
No et al (2002)	bacterium	B. cereus	MIC	500,000	72	1	2	250,000
No et al (2002)	bacterium	B. cereus	HONEC	1,000,000	72	1	1	1,000,000
Tsai et al (2002)	bacterium	B. cereus	MIC	200,000	48	1	2	100,000
Tsai et al (2002)	bacterium	B. cereus	MIC	200,000	48	1	2	100,000
Tsai et al (2002)	bacterium	B. cereus	MIC	1,000,000	48	1	2	500,000
Tsai et al (2002)	bacterium	B. cereus	MIC	500,000	48	1	2	250,000
Tsai et al (2002)	bacterium	B. cereus	MIC	1,000,000	48	1	2	500,000
Tsai et al (2002)	bacterium	B. cereus	MIC	1,000,000	48	1	2	500,000
No et al (2002)	bacterium	B. megaterium	MIC	800,000	72	1	2	400,000
No et al (2002)	bacterium	B. megaterium	MIC	500,000	72	1	2	250,000
No et al (2002)	bacterium	B. megaterium	MIC	800,000	72	1	2	400,000
No et al (2002)	bacterium	B. megaterium	MIC	500,000	72	1	2	250,000
No et al (2002)	bacterium	B. megaterium	MIC	500,000	72	1	2	250,000
No et al (2002)	bacterium	B. megaterium	MIC	800,000	72	1	2	400,000
Tsai et al (2002)	bacterium	L. monocytogenes	MIC	100,000	48	1	2	50,000
Tsai et al (2002)	bacterium	L. monocytogenes	MIC	150,000	48	1	2	75,000
Tsai et al (2002)	bacterium	L. monocytogenes	MIC	150,000	48	1	2	75,000
Tsai et al (2002)	bacterium	L. monocytogenes	MIC	150,000	48	1	2	75,000
Tsai et al (2002)	bacterium	L. monocytogenes	MIC	150,000	48	1	2	75,000
Tsai et al (2002)	bacterium	L. monocytogenes	MIC	150,000	48	1	2	75,000
No et al (2002)	bacterium	L. monocytogenes	MIC	1,000,000	72	1	2	500,000
No et al (2002)	bacterium	L. monocytogenes	HONEC	1,000,000	72	1	1	1,000,000
No et al (2002)	bacterium	L. monocytogenes	MIC	800,000	72	1	2	400,000
No et al (2002)	bacterium	L. monocytogenes	MIC	800,000	72	1	2	400,000
No et al (2002)	bacterium	L. monocytogenes	MIC	800,000	72	1	2	400,000
No et al (2002)	bacterium	L. monocytogenes	MIC	1,000,000	72	1	2	500,000
Tsai et al (2002)	fungi	F. oxysporum	MIC	500,000	168	1	2	250,000
Tsai et al (2002)	fungi	F. oxysporum	MIC	500,000	168	1	2	250,000
Tsai et al (2002)	fungi	F. oxysporum	MIC	1,000,000	168	1	2	500,000
Tsai et al (2002)	fungi	F. oxysporum	MIC	500,000	168	1	2	250,000
Tsai et al (2002)	fungi	F. oxysporum	HONEC	2,000,000	168	1	1	2,000,000
Tsai et al (2002)	fungi	F. oxysporum	HONEC	2,000,000	168	1	1	2,000,000

Table S4: Data for soil toxicity of chitosan (cont.)

Reference	Taxonomy	Test organisms	Ecotoxical endpoint	Concentration (μg/kg)	Exposure time (h)	AF-time	AF-NOEC	species sensitivity (μg/L) = concentratioNAF
Tsai et al (2002)	fungi	A. fumigatus	HONEC	2,000,000	168	1	1	2,000,000
Tsai et al (2002)	fungi	A. fumigatus	HONEC	2,000,000	168	1	1	2,000,000
Tsai et al (2002)	fungi	A. fumigatus	HONEC	2,000,000	168	1	1	2,000,000
Tsai et al (2002)	fungi	A. fumigatus	HONEC	2,000,000	168	1	1	2,000,000
Tsai et al (2002)	fungi	A. fumigatus	HONEC	2,000,000	168	1	1	2,000,000
Tsai et al (2002)	fungi	A. fumigatus	HONEC	2,000,000	168	1	1	2,000,000
Tsai et al (2002)	fungi	A. parasiticus	HONEC	2,000,000	168	1	1	2,000,000
Tsai et al (2002)	fungi	A. parasiticus	HONEC	2,000,000	168	1	1	2,000,000
Tsai et al (2002)	fungi	A. parasiticus	HONEC	2,000,000	168	1	1	2,000,000
Tsai et al (2002)	fungi	A. parasiticus	HONEC	2,000,000	168	1	1	2,000,000
Tsai et al (2002)	fungi	A. parasiticus	HONEC	2,000,000	168	1	1	2,000,000
Tsai et al (2002)	fungi	A. parasiticus	HONEC	2,000,000	168	1	1	2,000,000

Table S5: Data for soil toxicity of HAP

Reference	Taxonomy	Test organisms	Ecotoxical endpoint	Concentration (μg/kg)	Exposure time (h)	AF-time	AF-NOEC	species sensitivity (μg/L) = concentratioNAF
Baskar et al (2016)	bacterium	K. pneumoniae	MIC	292,800	24	1	2	146,400

Table S6: Data for soil toxicity of PAN

Reference	Taxonomy	Test organisms	Ecotoxical endpoint	Concentration (μg/kg)	Exposure time (h)	AF-time	AF-NOEC	species sensitivity (μg/L) = concentratioNAF
Shi et al (2011)	bacterium	B. cereus	HONEC	32,500,000	18	10	1	7,552,000

Table S7: Summary of nanoparticle size and characterization in collected ecotoxicological data points

Nano Particle	*Particle Size (nm)*	*# data points*	*Morphology/Characterization[1]*	*Reference*
Chitosan	40	10	Agglomerated nanoparticles, shaped like snowflakes	(Qi et al., 2004)
Chitosan	54	1	Primary nanoparticles[2], perfect spherical shape	(Du et al., 2008)
Chitosan	200	1	Primary nanoparticles, round shape	(Hu et al., 2011)
Chitosan	340	1	Primary nanoparticles, round shape	(Hu et al., 2011)
HAP	11	1	Agglomerated nanoparticles, rod-shaped	(Pereira et al., 2017)
HAP	14	5	Primary nanoparticles, spherical	(Baskar et al., 2017)
HAP	15	1	Primary nanoparticles, dots	(Pujari-Palmer et al., 2017)
HAP	19	1	Agglomerated nanoparticles, rod-shaped	(Pereira et al., 2017)
HAP	60 x 1000 to 4000	1	Primary nanoparticles, fibers	(Pujari-Palmer et al., 2017)
HAP	70	1	Primary nanoparticles, elongated spheroid	(Li et al., 2010)
HAP	75 x 30	1	Primary nanoparticles, sheets	(Pujari-Palmer et al., 2017)
HAP	150	1	Primary nanoparticles, rod-shaped	(Zhao et al., 2013)
HAP	200 x 20	1	Primary nanoparticles, long rods	(Pujari-Palmer et al., 2017)
HAP	230	1	Primary nanoparticles, needle-shaped	(Zhao et al., 2013)
PAN	221	1	La_2O_3 nanoparticle-doped PAN nanofibers[3]	(He et al., 2016)
PAN	400	2	Ag/PAN hybrid nanofibers[3]	(Shi et al., 2011)

[1] Forms and morphologies shown in TEM/AFM images
[2] The status of nanoparticles in the solution is dispersive
[3] Size distribution/particle characterization only available for certain forms of nanoparticles/nanofibers

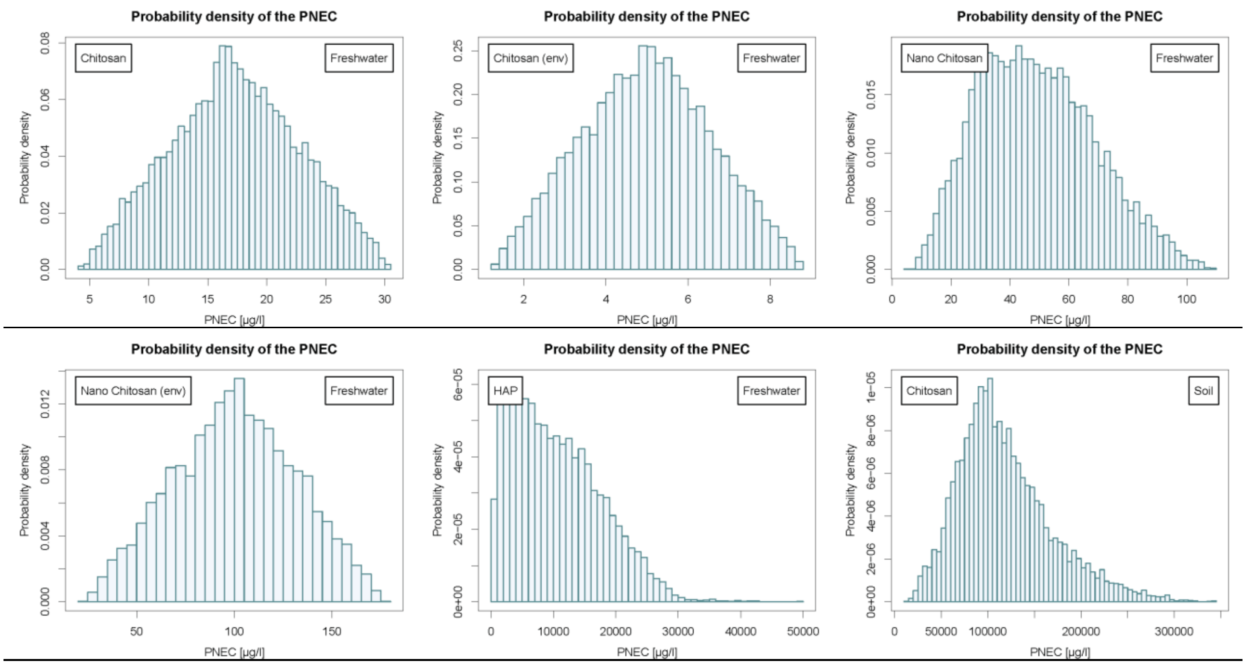

Figure S1: Predicted no-effect concentration (PNEC) distribution for chitosan, chitosan (env), nano chitosan, nano chitosan (env), HAP, and PAN

Table S8: Predicted no-effect concentrations (PNECs) of studied nanobiomaterials and other pollutants in freshwater

Material	*PNEC (ng/L)*	*Category*	*Reference*
Chitosan (env)	5,000	NanoBioMaterial	calculated
Nano Chitosan (env)	100,000	NanoBioMaterial	calculated
HAP	$1.1 * 10^7$	NanoBioMaterial	calculated
PAN	$3.0 * 10^9$	NanoBioMaterial	calculated
CNT	55,600	ENMs	(Coll et al., 2016)
Nano-TiO_2	15,700	ENMs	(Coll et al., 2016)
Fullerenes	3840	ENMs	(Coll et al., 2016)
Nano-ZnO	1000	ENMs	(Coll et al., 2016)
Nano-Ag	17	ENMs	(Coll et al., 2016)
Aspirin	61,000	Pharmaceuticals	(Jones et al., 2002)
Ibuprofen	9060	Pharmaceuticals	(Jones et al., 2002)
Estrogen	1060	Hormones	(Stuer-Lauridsen et al., 2000)
Doxycycline	300	Antibiotics	(Kümmerer & Henninger, 2003)
Amoxicillin	100	Antibiotics	(Kümmerer & Henninger, 2003)
Atrazine	100,000	Pesticides/POPs	(Sangchan et al., 2014)
Cu	50,000	Heavy metal	(Wang et al., 2010)
Triclosan	1550	Antimicrobial agent	(Capdevielle et al., 2008)
Pb & Cd	1000	Heavy metal	(Wang et al., 2010)
DBP	740	Plasticizer	(Slobodnik et al., 2012)
Hg	50	Heavy metal	(Wang et al., 2010)
Dichlorvos	1.9	Pesticides/POPs	(Sangchan et al., 2014)

Table S9: Median, mean, mode, minimum, maximum, 25th and 75th quantiles from predicted no-effect concentration (PNEC) distributions of chitosan in freshwater. All values in µg/l.

[µg/L]	*Chitosan*	*Nano-Chitosan*
Median	17	47
Mean	17	49
Mode	18	36
Min	4	8
25%	14	33
75%	21	62
Max	30	110

References

Baskar, K., Anusuya, T., & Devanand Venkatasubbu, G. (2017). Mechanistic investigation on microbial toxicity of nano hydroxyapatite on implant associated pathogens. *Materials Science and Engineering C*, *73*, 8–14. https://doi.org/10.1016/j.msec.2016.12.060

Capdevielle, M., Van Egmond, R., Whelan, M., Versteeg, D., Hofmann-Kamensky, M., Inauen, J., … Woltering, D. (2008). Consideration of exposure and species sensitivity of triclosan in the freshwater environment. *Integrated Environmental Assessment and Management*, *4*(1), 15–23. https://doi.org/10.1897/IEAM_2007-022.1

Coll, C., Notter, D., Gottschalk, F., Sun, T., Som, C., & Nowack, B. (2016). Probabilistic environmental risk assessment of five nanomaterials (nano-TiO2, nano-Ag, nano-ZnO, CNT, and fullerenes). *Nanotoxicology*, *10*(4), 436–444. https://doi.org/10.3109/17435390.2015.1073812

Costa, E. M., Silva, S., Pina, C., Tavaria, F. K., & Pintado, M. M. (2012). Evaluation and insights into chitosan antimicrobial activity against anaerobic oral pathogens. *Anaerobe*, *18*(3), 305–309. https://doi.org/10.1016/j.anaerobe.2012.04.009

Du, W. L., Xu, Y. L., Xu, Z. R., & Fan, C. L. (2008). Preparation, characterization and antibacterial properties against E. coli K88 of chitosan nanoparticle loaded copper ions. *Nanotechnology*, *19*(8), 0–5. https://doi.org/10.1088/0957-4484/19/8/085707

Fernandes, J. C., Tavaria, F. K., Soares, J. C., Ramos, Ó. S., João Monteiro, M., Pintado, M. E., & Xavier Malcata, F. (2008). Antimicrobial effects of chitosans and chitooligosaccharides, upon Staphylococcus aureus and Escherichia coli, in food model systems. *Food Microbiology*, *25*(7), 922–928. https://doi.org/10.1016/j.fm.2008.05.003

He, J., Wang, W., Shi, W., & Cui, F. (2016). La2O3nanoparticle/polyacrylonitrile nanofibers for bacterial inactivation based on phosphate control. *RSC Advances*, *6*(101), 99353–99360. https://doi.org/10.1039/c6ra22374e

Hu, Y.-L., Qi, W., Han, F., Shao, J.-Z., & Gao, J.-Q. (2011). Toxicity evaluation of biodegradable chitosan nanoparticles using a zebrafish embryo model. *International Journal of Nanomedicine*, *6*, 3351–3359. https://doi.org/10.2147/IJN.S25853

Jones, O. A. H., Voulvoulis, N., & Lester, J. N. (2002). Aquatic environmental assessment of the top 25 English prescription pharmaceuticals. *Water Research*, *36*(20), 5013–5022. https://doi.org/10.1016/S0043-1354(02)00227-0

Kümmerer, K., & Henninger, A. (2003). Promoting resistance by the emission of antibiotics from hospitals and

households into effluent. *Clinical Microbiology and Infection*, *9*(12), 1203–1214. https://doi.org/10.1111/j.1469-0691.2003.00739.x

Li, Y., Ho, J., & Ooi, C. P. (2010). Antibacterial efficacy and cytotoxicity studies of copper (II) and titanium (IV) substituted hydroxyapatite nanoparticles. *Materials Science and Engineering C*, *30*(8), 1137–1144. https://doi.org/10.1016/j.msec.2010.06.011

No, H. ., Park, N. ., Lee, S. ., & Meyers, S. (2002). Antibacterial activity of chitosans and chitosanoligomers with different molecular weights. *Int. J.FoodMicrobiol*, *74*, 65–72.

Pereira, F. F., Paris, E. C., Bresolin, J. D., Foschini, M. M., Ferreira, M. D., & Corrêa, D. S. (2017). Investigation of nanotoxicological effects of nanostructured hydroxyapatite to microalgae Pseudokirchneriella subcapitata. *Ecotoxicology and Environmental Safety*, *144*(June), 138–147. https://doi.org/10.1016/j.ecoenv.2017.06.008

Pujari-Palmer, S., Lu, X., & Ott, M. K. (2017). The Influence of Hydroxyapatite Nanoparticle Morphology on Embryonic Development in a Zebrafish Exposure Model. *Nanomaterials*, *7*(4), 89. https://doi.org/10.3390/nano7040089

Qi, L., Xu, Z., Jiang, X., Hu, C., & Zou, X. (2004). Preparation and antibacterial activity of chitosan nanoparticles. *Carbohydrate Research*, *339*(16), 2693–2700. https://doi.org/10.1016/j.carres.2004.09.007

Rizzo, L., Di Gennaro, A., Gallo, M., & Belgiorno, V. (2008). Coagulation/chlorination of surface water: A comparison between chitosan and metal salts. *Separation and Purification Technology*, *62*(1), 79–85. https://doi.org/10.1016/j.seppur.2007.12.020

Sadeghi, A. M. M., Dorkoosh, F. A., Avadi, M. R., Saadat, P., Rafiee-Tehrani, M., & Junginger, H. E. (2008). Preparation, characterization and antibacterial activities of chitosan, N-trimethyl chitosan (TMC) and N-diethylmethyl chitosan (DEMC) nanoparticles loaded with insulin using both the ionotropic gelation and polyelectrolyte complexation methods. *International Journal of Pharmaceutics*, *355*(1–2), 299–306. https://doi.org/10.1016/j.ijpharm.2007.11.052

Sangchan, W., Bannwarth, M., Ingwersen, J., Hugenschmidt, C., Schwadorf, K., Thavornyutikarn, P., … Streck, T. (2014). Monitoring and risk assessment of pesticides in a tropical river of an agricultural watershed in northern Thailand. *Environmental Monitoring and Assessment*, *186*(2), 1083–1099. https://doi.org/10.1007/s10661-013-3440-8

Shi, Q., Vitchuli, N., Nowak, J., Caldwell, J. M., Breidt, F., Bourham, M., … McCord, M. (2011). Durable antibacterial Ag/polyacrylonitrile (Ag/PAN) hybrid nanofibers prepared by atmospheric plasma treatment and electrospinning. *European Polymer Journal*, *47*(7), 1402–1409. https://doi.org/10.1016/j.eurpolymj.2011.04.002

Sigma-Aldrich. (2016). Safety Data Sheet, 1–7.

Slobodnik, J., Mrafkova, L., Carere, M., Ferrara, F., Pennelli, B., Schüürmann, G., & von der Ohe, P. C. (2012). Identification of river basin specific pollutants and derivation of environmental quality standards: A case study in the Slovak Republic. *TrAC - Trends in Analytical Chemistry*, *41*, 133–145. https://doi.org/10.1016/j.trac.2012.08.008

Stuer-Lauridsen, F., Birkved, M., Hansen, L. P., Holten Lützhøft, H.-C., & Halling-Sørensen, B. (2000). Environmental risk assessment of human pharmaceuticals in Denmark after normal therapeutic use. *Chemosphere*, *40*(7), 783–793. https://doi.org/10.1016/S0045-6535(99)00453-1

Tsai, G. J., Su, W. H., Chen, H. C., & Pan, C. L. (2002). Antimicrobial activity of shrimp chitin and chitosan from different treatments and applications of fish preservation. *Fisheries Science*, *68*(1), 170–177. https://doi.org/10.1046/j.1444-2906.2002.00404.x

Wang, J., Chen, S., & Xia, T. (2010). Environmental risk assessment of heavy metals in Bohai Sea, North China. *Procedia Environmental Sciences*, *2*, 1632–1642. https://doi.org/10.1016/j.proenv.2010.10.174

Zhao, X., Ong, K. J., Ede, J. D., Stafford, J. L., Ng, K. W., Goss, G. G., & Loo, S. C. J. (2013). Evaluating the toxicity of hydroxyapatite nanoparticles in catfish cells and zebrafish embryos. *Small*, *9*(9–10), 1734–1741. https://doi.org/10.1002/smll.201200639
